# Systemic Identification and Functional Characterization of Common in Fungal Extracellular Membrane Proteins in *Lasiodiplodia theobromae*

**DOI:** 10.3389/fpls.2021.804696

**Published:** 2021-12-20

**Authors:** Junbo Peng, Linna Wu, Wei Zhang, Qi Zhang, Qikai Xing, Xuncheng Wang, Xinghong Li, Jiye Yan

**Affiliations:** Beijing Key Laboratory of Environment Friendly Management on Fruit Diseases and Pests in North China, Institute of Plant Protection, Beijing Academy of Agriculture and Forestry Sciences, Beijing, China

**Keywords:** *Lasiodiplodia theobromae*, CFEM domain, cell death, immune response, temperature

## Abstract

Plant pathogenic fungi deploy secreted proteins into apoplastic space or intracellular lumen to promote successful infections during plant-pathogen interactions. In the present study, fourteen CFEM domain-containing proteins were systemically identified in *Lasiodiplodia theobromae* and eight of them were functionally characterized. All eight proteins were confirmed to be secreted into extracellular space by a yeast signal peptide trapping system. The transcriptional levels of most CFEM genes, except for *LtCFEM2* and *LtCFEM6*, were significantly elevated during infection. In addition, almost all *LtCFEM* genes, apart from *LtCFEM2*, *LtCFEM3*, and *LtCFEM6*, were transcriptionally up-regulated at 35°C in contrast to that at 25°C and 30°C. As two elicitors, LtCFEM1 induced local yellowish phenotype and LtCFEM4 triggered cell death in *Nicotiana benthamiana* leaves. Furthermore, these proteins displayed distinct subcellular localizations when expressed transiently in *N. benthamiana*. Moreover, two genes, *LtCFEM7* and *LtCFEM8*, were found to be spliced alternatively by RT-PCR and sequencing. Therefore, our data suggest that LtCFEM proteins play important roles in multiple aspects, including pathogenicity and plant immune response, which will enhance our understanding of the sophisticated pathogenic mechanisms of plant opportunistic pathogen *L. theobromae*.

## Introduction

The ascomycetous fungus *L. theobromae* and other related species in Botryosphaeriaceae are economically important and destructive fungal pathogens (Úrbez-Torres et al., 2008; Félix et al., 2019). They are well-known pathogens causing various cankers, dieback, and fruit and root rots in almost 500 plant species globally (Úrbez-Torres et al., 2008; Paolinelli-Alfonso et al., 2016; Marsberg et al., 2017). Members of the Botryosphaeriaceae including *L. theobromae* are endophytes and latent pathogens that survive inside plant vascular tissues for a certain period without showing any symptoms at appropriate environmental conditions (Chethana et al., 2016; Yan et al., 2018). However, in the presence of external stimuli from changing environmental conditions inside or outside hosts, some fungi can change their lifestyles from endophytic to pathogenic (Harishchandra et al., 2020). Because of this lifestyle transition, these species are regarded as plant opportunistic fungal pathogens (Chethana et al., 2016). The underlying mechanisms of the lifestyle transition caused by external stimuli are yet unrevealed.

The grapevine canker disease caused by *L. theobromae* has become one of the most notorious fruit diseases responsible for a considerable loss of yield to the grape industry worldwide each year (Yan et al., 2013, 2018; Harishchandra et al., 2020). Owing to the significant economic losses caused by *L. theobromae*, research on the grapevine and *L. theobromae* interactions have received more attention in recent years, and thereby, a series of significant progress have been achieved. To date, a large number of pathogenicity related factors, including secondary metabolites, plant cell wall degrading enzymes, and phytotoxins have been identified and characterized for *L. theobromae* (Andolfi et al., 2011; Yan et al., 2018; Chethana et al., 2020; Aluthmuhandiram et al., 2021). Endopolygalacturonase LtEPG1 was reported as an important virulence factor during *L. theobromae* infection and triggered cell death in *Nicotiana benthamiana* (Chethana et al., 2020). Cao et al. (2020) revealed that *L. theobromae* degrade benzo[a]pyrene efficiently as its sole carbon source, implying the possibility of exploring *L. theobromae* as a potential strain for the removal of polycyclic aromatic hydrocarbons in contaminated environments. The advent of high throughput technologies has allowed for a better understanding of the sophisticated plant-pathogen interactions of grapevine diseases. Moreover, comparative genomics and transcriptomic analyses on three Botryosphaeriaceous species, *L. theobromae, Botryosphaeria dothidea*, and *Neofusicoccum parvum* showed an expansion of gene families associated with cell wall degradation, nutrient uptake, secondary metabolism and membrane transport and revealed their adaptations to opportunistic infections (Yan et al., 2018). Furthermore, transcriptome analyses exhibited that hundreds of genes involved in plant-pathogen interactions, hormone signal transduction and phenylpropanoid biosynthesis pathways were highly expressed at the infectious stages, suggesting innate immunity, phytohormone signaling and many phenylpropanoid compounds are involved in the response of grapevine against *L. theobromae* infection (Paolinelli-Alfonso et al., 2016; Zhang et al., 2019). Moreover, a multi-omics analysis of *L. theobromae* identified hundreds of pathogenicity-related genes and analyzed their expression under different temperatures (Félix et al., 2019). These studies provide novel insights into the molecular mechanisms of grapevine-*L. theobromae* interactions that are valuable for genetic improvement of grapevine resistance against plant pathogens.

CFEM domain (**C**ommon in **F**ungal **E**xtracellular **M**embrane, Pfam accession PF05730) is considered to be unique to fungi and contains a consensus of eight characteristically spaced cysteine residues (Vaknin et al., 2014; Sabnam and Barman, 2017; Zhu et al., 2017; Gong et al., 2020). Generally, this domain consists of about 60 amino acids and is characterized typically by the following sequence: PxC[A/G]x2Cx8–12Cx1–3[x/T]Dx2–5CxCx9–14Cx3–4Cx15–16C, where “x” is any residue with a range indicated. Besides the C, other residues, including P, A and T/D, are also relatively conservative (Kulkarni et al., 2003; Zhang et al., 2015; Sabnam and Barman, 2017). Usually, the CFEM domain-containing proteins (hereinafter referred to as CFEM proteins) contains one or multiple copies of the CFEM domain, normally with one copy at their N terminus. Besides, one signal peptide, along with transmembrane spans or glycosylphosphatidylinositol (GPI) anchor, are also present in this kind of protein (Kulkarni et al., 2003). Apart from that, it seems that no other typical domains are characterized in these proteins. Additionally, some CFEM proteins are localized in the cell membrane or are surface antigens (Zhu et al., 1996; Moukadiri et al., 1997; DeZwaan et al., 1999; Lamarre et al., 2000). The first CFEM protein identified is MoACI1, which is an adenylate cyclase (MAC1)-interacting protein, identified in *Magnaporthe oryzae*. This protein contains one signal peptide, one CFEM domain, and one GPI anchor site (Kulkarni et al., 2003). Despite the limited research history on CFEM proteins, they have been identified intensively in model pathogenic fungi including *M. oryzae* (DeZwaan et al., 1999; Kulkarni et al., 2003), *Botrytis cinerea* (Zhu et al., 2017), *Candida albicans* (Lamarre et al., 2000; Chen et al., 2011; Okamoto-Shibayama et al., 2014), and *Colletotrichum graminicola* (Gong et al., 2020). A total of 20 CFEM proteins have been identified in *M. oryzae* and some of these proteins such as CFEM-GPCRs Pth11 and WISH were found to be necessary for appressorium formation and fungal pathogenesis (DeZwaan et al., 1999; Zhang et al., 2015; Sabnam and Barman, 2017). Furthermore, nine CFEM proteins were identified in *Candida albicans* (Zhang et al., 2015). Among them, the Csa2 was reported to be involved in the utilization of hemoglobin as an iron source by the hyphal form of *C. albicans*, and CSA1 was reported to be associated with biofilm development (Perez et al., 2006; Okamoto-Shibayama et al., 2014). Moreover, as described in *B. cinerea*, BcCFEM1 affected fungal virulence, conidiation, oxidative and osmotic stress tolerance (Zhu et al., 2017). Besides, CFEM proteins are documented in non-pathogenic model fungi, such as *Saccharomyces cerevisiae*. It has been observed that CFEM protein, CCW14 was found to be involved in cell wall biogenesis and played an important role in maintaining cell wall integrity and stability in *S. cerevisiae* (Moukadiri et al., 1997; Mrša et al., 1999; Mrša and Tanner, 1999). These studies revealed that it’s important to uncover the multiple biological functions of CFEM proteins in microorganisms.

Here, we systemically identified a total of 14 CFEM proteins in *L. theobromae* by bioinformatic analyses. Among them, eight proteins were assumed as effectors as defined by their structural features and subsequently analyzed for biological function. The real time quantitative PCR (qRT-PCR) data revealed the transcription of these *LtCFEM* genes were mediated by the temperature factor. Interestingly, we found that the transcripts of *LtCFEM7* and *LtCFEM8* could be alternatively spliced, indicating that both genes may function in multiple forms, including proteins and RNAs. Moreover, transient expression showed that LtCFEM1 induced local yellowish phenotype and LtCFEM4 triggered cell death in *N. benthamiana* leave, suggesting the involvement of LtCFEM1 and LtCFEM4 proteins in immune responses to biotic stress. Our founding not only lay a foundation for analyzing the opportunistic pathogenicity mechanisms of *Botryosphaeriaceous* family pathogen in-depth, but also call more attention to establishing a set of effectively controlling methods against grapevine canker disease in response to the rising global temperature.

## Materials and Methods

### Strains, Plant Materials and Cultural Conditions

The *L. theobromae* wild type strain CSS-01s was cultured on the complete medium (6 g yeast extract, 3 g casein acid hydrolyzate, 3 g casein enzymatic hydrolyzate, and 10 g sucrose per liter) at 25°C. Yeast strain YTK12 and resultant yeast transformants were cultured on CMD-W (0.67% yeast nitrogen without amino acids, 0.075% tryptophan dropout supplement, 2% sucrose, 0.1% glucose, and 2% agar), and YPRAA (1% yeast extract, 2% peptone, 2% raffinose, 2 μg/mL antimycin A, and 2% agar) media. The *Agrobacterium tumefaciens* strain GV3101 and *Escherichia coli* (*E*. *coli*) strain BL21 were cultured on Luria Bertani medium (5 g yeast extract, 10 g tryptone, 10 g NaCl per liter, and 16 g agar was added per liter for plates). The *N. benthamiana* was grown inside a growth chamber at 25°C under a 14 h: 10 h light: dark photoperiod. Healthy green shoots of *Vitis vinifera* cv. “Summer Black” were obtained from Xiangyi field vineyard in Shunyi, China. Antibiotics were added into LB medium, with the following final concentrations, 50 μg/mL ampicillin, 50 μg/mL kanamycin, and 50 μg/mL rifampin.

### Identification of Common in Fungal Extracellular Membrane Protein Repertoire in *Lasiodiplodia theobromae*

To identify the CFEM proteins in *L. theobromae*, BLASTP searches against the *L. theobromae* proteome database were performed using the amino acid sequence of ACI1 CFEM domain from *M. oryzae* as the query sequence with a threshold of E-value < 5e–5. Three LtCFEM proteins LtCFEM3, LtCFEM7, and LtCFEM8 were identified upon the BLASTP searches. To thoroughly fish all the LtCFEM proteins in *L. theobromae*, we in turn performed further BLASTP searches against the *L. theobromae* proteome database using the amino acid sequences of LtCFEM3, LtCFEM7, and LtCFEM8 as the query. Total LtCFEM protein obtained was further examined for the presence of CFEM domain using the PFAM program.^1^

### Grapevine Inoculation Tests for Transcription Profiling at Different Hours Post Inoculation

The inoculation tests were performed on detached *Vitis vinifera* cv. “Summer Black” green shoots with methods described by Harishchandra et al. (2020). Green grapevine shoots were inoculated with mycelium plugs (5 mm in diameter) cut with a cork-borer and then wrapped up with the parafilm. Afterward, the inoculated grapevines shoots were maintained under an alternating dark/light cycle in a controlled growth chamber with 80% humidity and 25°C. The infected tissues were collected at different hours post inoculation (hpi) for subsequent RNA extractions and transcriptional investigation. The experiment was conducted independently three times, and eight shoots were used as biological replicates for each time.

### Culture Condition for Transcription Investigation Under Different Temperatures

The relative transcript level of *LtCFEM* genes under different temperatures was performed with the method as follows. A total of 15 mycelial plugs with 10 mm in diameter were incubated in liquid complete media, with or without 0.5% grapevine shoot powder supplements at different temperatures (25°C, 30°C, and 35°C) for 36 h, followed by mycelium collection, RNA extractions, and gene expression investigation.

### RNA Extraction and Real Time Quantitative PCR

Total RNA was extracted using the TRIzol reagent (Invitrogen, California, America). Isolated RNA was re-transcribed into cDNA with a TransScript^®^ One-Step gDNA Removal and cDNA Synthesis SuperMix kit (TransGen Biotech, Beijing, China). The qRT-PCR was performed in an ABI 7500 Real-Time system (Applied Biosystems, Waltham, MA, United States) and conducted in 20 uL volumes comprised of 10 μL RealStar Green Fast Mixture with ROX II (GenStar Biosolutions, Beijing, China), 1.0 μL cDNA, 0.2 μM primer, and 8.2 μL sterile ddH_2_O. The PCR program was progressed as follows: denaturation at 95°C for 2 min, followed by 40 cycles of 95°C for 15 s and 60°C for 30 s. The a*ctin* gene was used as the internal control. Expression data were normalized by *actin* gene and calibrated against the transcript levels at 12 hpi. The relative abundance of transcripts was calculated using the 2^–ΔΔCT^ method (Livak and Schmittgen, 2001). All the experiments were repeated at least twice independently with three replicates each. All the primers used for qRT-PCR assays were listed in Supplementary Table 1.

### Functional Validation of the LtCFEM Proteins Signal Peptides

Functional validation of the signal peptides of all LtCFEM proteins was conducted with an elegant yeast signal peptide trapping system (Jacobs et al., 1997; Gu et al., 2011; Fang et al., 2016). In this system, the signal peptide trapping vector *pSUC2T7M13ORI* (*pSUC2*) carries a truncated invertase gene *SUC2* which lacks its initiating methionine codon and signal peptide coding sequence. The predicted coding sequences of LtCFEM protein signal peptides were amplified with specific primer pairs (Supplementary Table 1) and then cloned into the *pSUC2* vector. The fusion construct was confirmed by sequencing and then transformed into invertase secretion-defective yeast strain YTK12. The resultant yeast transformants were streaked on CMD-W and YPRAA media to test for the invertase secretion abilities. Yeast cells expressing Avr1b from *P. sojae* and Mg87 from *M. oryzae* were used as positive and negative controls, respectively.

### *Agrobacterium tumefaciens*-Mediated Transient Expression

The full-length ORFs or truncated ORFs without signal peptide of *LtCFEM* genes were amplified with primer pairs listed in Supplementary Table 1 and cloned into an expression plasmid driven by cauliflower mosaic virus 35S promoter. The resultant fusion constructs were transformed into *A. tumefaciens* GV3101 using a freeze-thaw method, respectively (Fang et al., 2016). The resulting positive transformants were confirmed by PCR. After overnight culturing, *A. tumefaciens* were harvested by centrifugation at 4000 rpm for 10 min, and washed three times with sterile ddH_2_O. Subsequently, the *A. tumefaciens* were resuspended in infiltration buffer [10 mM MES, 2-(N-morpholino) ethanesulfonic acid, pH 5.7, 10 mM MgCl_2_, and 150 μM acetosyringone] with an optical density of 0.8 at 600 nm. After standing for 3 h at room temperature, the *A. tumefaciens* suspensions were infiltrated into 4-week-old *N. benthamiana* leaves with a needleless syringe. *A. tumefaciens* expressing the pro-apoptotic BAX or INF1 protein was infiltrated as positive control and *A. tumefaciens* expressing GFP tag was used as negative control. The treated leaves were collected 3 d post agroinfiltration and fluorescence was detected with a Zeiss LSM710 confocal laser-scanning microscope at 488 nm excitation for subcellular localization tests. Additionally, the infiltrated leaves were photographed 9 d post agroinfiltration.

### Measurement of Ion Leakage of *Nicotiana benthamiana* Leaf Discs

The cell death of injected *N. benthamiana* leaves was assessed by measuring the ion leakage from leaf discs with minor modifications (Mittler et al., 1999; Hwang and Hwang, 2011; Yu et al., 2012; Fang et al., 2016). Briefly, a total of five leaf discs (15 mm in diameter) were floated abaxial side up on 10 mL distilled water at room temperature for 3 h. Afterward, the conductivity of the bathing solution (A) was measured with a conductivity meter (Starter 3100C, OHAUS, Pine Brook, United States). Subsequently, the leaf discs were re-placed in the bathing solution and boiled in sealed tubes for 25 min. After the bathing solution cooled to room temperature, the conductivity (B) was measured again. For each measurement, the ion leakage was described as leakage percentage calculated as (A/B) × 100.

### Plasmolysis Experiments

Plasmolysis experiments were performed with agroinfiltrated *N. benthamiana* leaves (cut it to small squares) immersed in 0.05 M MES buffer with the plasmolyticum (0.75 M sorbitol) for 15 min. Subsequently, the samples were examined with a Zeiss LSM 710 confocal laser scanning microscope. The GFP was excited at the wavelength of 488 nm. Images were progressed using the Zeiss LSM 710 microscope and adobe photoshop software.

### Sequence Homology Analyses

The signal peptides of LtCFEM proteins were predicted with SignalP 5.0 server with default parameter settings.^2^ The CFEM domain of each LtCFEM protein was identified through the BLASTP search against the Pfam database using their amino acid sequences as a query. The transmembrane domains were predicted using the TMHMM tool.^3^ The amino acid sequence of CFEM domain was extracted from its full-length protein, and multiple sequence alignments were generated using ClustalX. Identical amino acids were shaded using Jalview and further analyzed with WebLogo Server.^4^

### Phylogenetic Analysis

The phylogenetic tree was constructed using the neighbor-joining method with MEGA7 program, and bootstrap analyses were conducted using the P-distance model with 2000 replications. The amino acid sequences of MoCsa2 from *C. albicans* and MoACI1 obtained from *M. oryzae* were obtained through BLASTP programs against the NCBI database with LtCFEM1 as the query sequence.

### Structure Prediction

The structures of all LtCFEM proteins, CaCsa2 from *C. albicans* and MoACI1 from *M. oryzae* were modeled with Phyre2^5^ tools. The amino acid sequences were blasted with PSI-Blast for homologies, and the Hidden Markov Model structures were built.

### Statistical Analyses

All the data were analyzed using the one-way analysis of variance (ANOVA) and least significant difference (LSD) tests with SPSS Statistics 25.0. The values are represented as means ± standard deviation.

## Results

### Identification and Structural Analyses of Common in Fungal Extracellular Membrane Proteins in *Lasiodiplodia theobromae*

**FIGURE 1:**
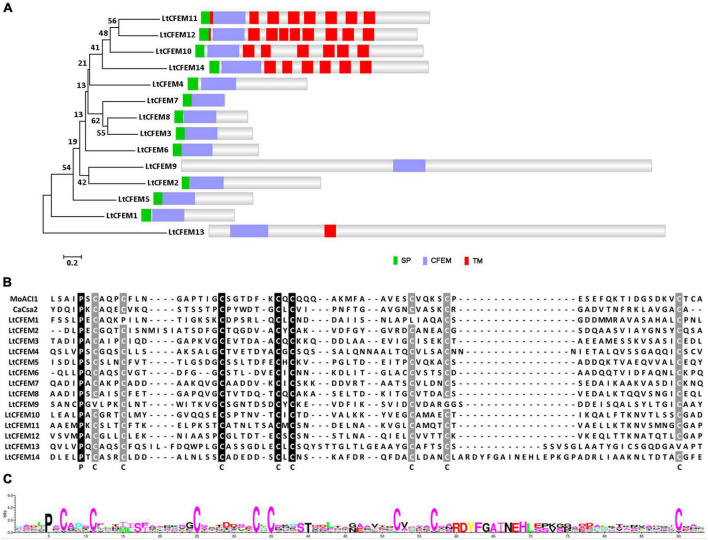
Structural and phylogenic analyses of CFEM proteins in *Lasiodiplodia theobromae*. **(A)** Structural and phylogenic analyses of CFEM proteins. The amino acid sequences of total CFEM proteins were sourced from NCBI and subsequently used to generate the phylogenetic tree using MEGA7 with the neighbor-joining method, 2,000 replicates. Bootstrap percentage support for each branch is indicated at the nodes. The signal peptides of CFEM proteins were predicted with SignalP 5.0 server with default parameter settings (http://www.cbs.dtu.dk/services/SignalP/). The number and location of CFEM domain were predicted using BLASTP programs against Pfam database with each amino acid sequence as the query. The transmembrane domain was predicted using the TMHMM tool (http://www.cbs.dtu.dk/services/TMHMM/). SP, Signal Peptide. CFEM, CFEM domain. TM, Transmembrane Domain. **(B)** Multiple sequence alignments of CFEM domains. CFEM domains of Csa2 from *Candida albicans*, and ACI1from *Magnaporthe oryzae* were used as references. Amino acid sequences were aligned using ClustalX2 Program and the alignments were edited with Jalview software. The conserved amino acid is highlighted in black and gray, and marked with the corresponding letters. **(C)** WebLogo analyses of CFEM domains. Letters of prominent size indicated amino acids with a higher conservation level.

To identify the CFEM proteins in *L. theobromae*, BLASTP searches were performed with the ACI1 amino acid sequence from *M. oryzae* as the query sequence. Three LtCFEM proteins were firstly identified based on the BLASTP searches. To obtain all the LtCFEM proteins in *L. theobromae*, we performed further BLASTP searches against the *L. theobromae* proteome database using the amino acid sequences of three identified CFEM proteins as the query, respectively. Finally, a total of 14 CFEM proteins were identified and these proteins were named LtCFEM1 to LtCFEM14, respectively. Among them, one protein LtCFEM9 was predicted without signal peptide based on SignalP 5.0 analysis. Furthermore, five of them were predicted to contain one or several transmembrane domains upon BLASTP search against the Pfam database. The remaining eight proteins contained one signal peptide and one typical CFEM domain near the N-terminus. Based on their amino acid sequences, a phylogram was also generated for all CFEM proteins in *L. theobromae*. The phylogram showed that proteins with one or more transmembrane domains, except for LtCFEM13, were grouped. In contrast, another clade, except for LtCFEM13, did not contain a transmembrane domain (Figure 1A).

To further determine the conserved amino acids of the CFEM domain, sequences of CFEM domains of proteins LtCFEM1 to LtCFEM14 were aligned with MEGA7. Two well documented CFEM proteins, Csa2 from *C. albicans* and ACI1 from *M. oryzae* were used as reference subjects (Kulkarni et al., 2003; Okamoto-Shibayama et al., 2014). These completely aligned proteins, except for LtCFEM1, LtCFEM9, and LtCFEM13, contained approximately 70 amino acids in length and 8 conserved cysteines, which could form four disulfide bonds to stabilize the whole structure of the CFEM domain. Moreover, one proline located at one amino acid upstream the first conserved cysteine was stringently conserved among all the CFEM domains (Figure 1B).

WebLogo analysis further confirmed that proline and cysteines were highly conserved in the CFEM domains of these proteins (Figure 1C). Because of the secreted ability of effectors, proteins with a typical signal peptide and without transmembrane domains, named LtCFEM1 to LtCFEM8, were selected for further functional exploration.

**FIGURE 2:**
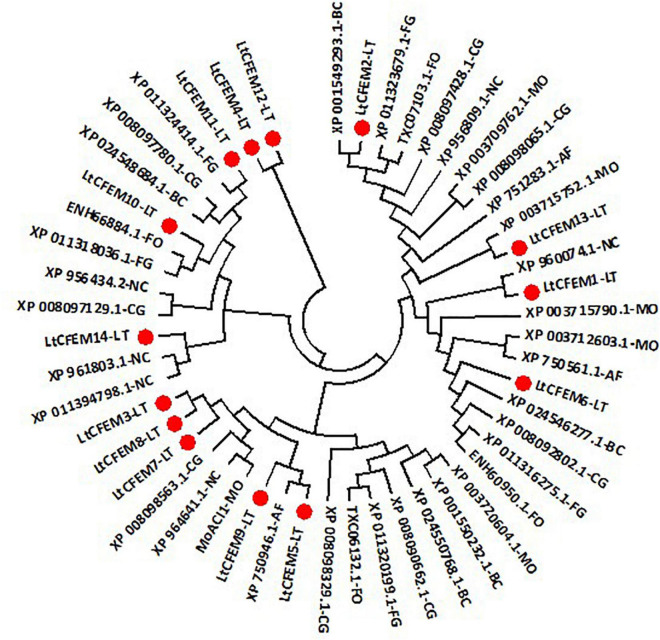
A neighbor-joining phylogenetic tree of LtCFEM proteins and CFEM proteins from other fungi. The amino acid sequences were obtained through BLASTP searches against the genome database of related fungi with the amino acid sequences of each LtCFEM protein as the query. The phylogenic tree was constructed using MEGA7 with the similar method described in Figure 1. MO, *Magnaporthe oryzae*; BC, *Botrytis cinerea*; FG, *Fusarium graminearum*; CG, *Colletotrichum graminicola*; FO, *Fusarium oxysporum*; NC, *Neurospora crassa*; AF, *Aspergillus fumigatus*; LT, *Lasiodiplodia theobromae*.

To analyze their relatedness with other CFEM proteins from plant pathogenic and non-pathogenic model fungi including *M. oryzae*, *Fusarium oxysporum*, *and N. crassa*, we constructed a phylogenic tree based on their amino acid sequences. The phylogram displayed that CFEM proteins were widely distributed across pathogenic and non-pathogenic fungi (Figure 2). Moreover, CFEM proteins containing predicted transmembrane domains were clustered closer, suggesting that the evolution process may be closely related to the protein structure and biological function.

### Helical-Basket Shape Detected in the Predicted Structure of Common in Fungal Extracellular Membrane Proteins

As Phyre2 has been used widely for predicting the three-dimensional (3D) structure of proteins, we used them to determine the structures of all eight CFEM proteins and analyze their homologies. The predicted models showed that amino acid sequences of all the CFEM proteins have high similarity with the protein Csa2 from *C. albicans*, which is the first identified CFEM protein with a detailed 3D crystal structure (Nasser et al., 2016). In addition, all CFEM domains were modeled with over 97% confidence to Csa2.

**FIGURE 3:**
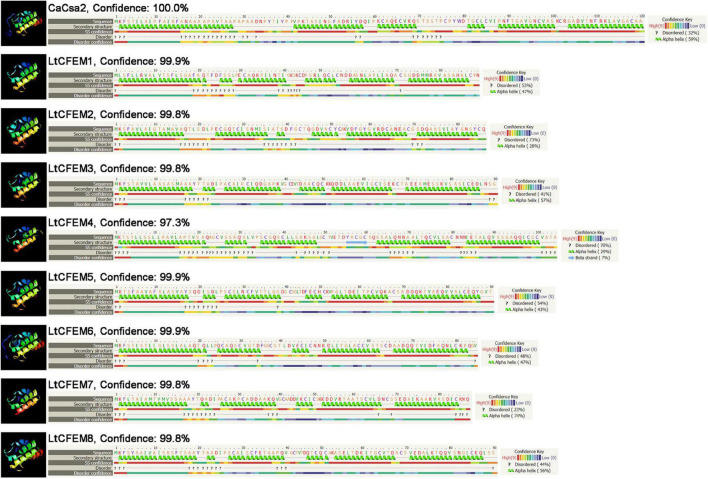
Modeling structural analysis of LtCFEM1 to LtCFEM8 in *Lasiodiplodia theobromae* with Phyre2 server (http://www.sbg.bio.ic.ac.uk/phyre2/html/page. cgi?id=index) and RoseTTAFold server (https://robetta.bakerlab.org/submit.php). The amino acid residues of these effectors were modeled to crystal structure of the CFEM domain in Csa2 from *Candida albicans* with more than 95% confidence. The amino acid sequence of each protein was indicated in the top line. The sequence was on the next line with residues colored according to property-based scheme: yellow (A, S, T, G, P: small/polar), green (M, I, L, V: hydrophobic), red (K, R, E, N, D, H, Q: charged) and purple (W, Y, F, C: aromatic and cysteine). Green helices represent α-helix, blue arrows indicate β-strands, and faint lines indicate coils.

The structural comparison indicates the CFEM domain of LtCFEM6 comprised five α-helices to form a compact helical-basket shape with an elongated handle, which was similarly characterized in Csa2. Unlike LtCFEM6, other proteins were also predicted to contain a helical basket-like structure but without an elongated handle. Similar to Csa2, the CFEM domain of LtCFEM1, LtCFEM2, LtCFEM3, LtCFEM5, LtCFEM7, and LtCFEM8 presumably contained six α-helices and LtCFEM4, however, carried five α-helices and one β-strand (Figure 3).

### Expression Profiles of Eight Putative *LtCFEM* Genes During *Lasiodiplodia theobromae* Infection on Grapevines

**FIGURE 4:**
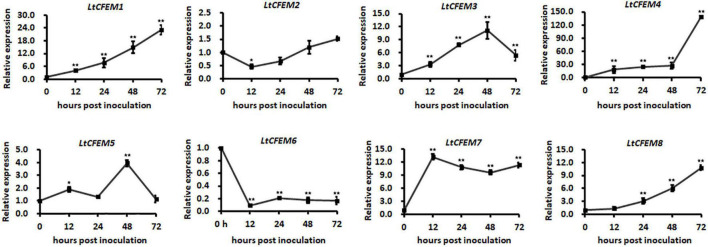
Expression profiles of eight *LtCFEM* genes during *Lasiodiplodia theobromae* infection of grapevines. The grapevine tissues infected by wild type CSS-01 s were harvested at 0, 12, 24, 48, and 72 h post inoculation (hpi) for RNA extractions. The isolated RNA was reverse transcribed into cDNA for *LtCFEM* gene expression analysis using quantitative real time reverse transcription-polymerase chain reaction assay. Relative transcription level of each *LtCFEM* gene was calculated using the 2^–ΔΔCT^ method. Relative transcript levels of *LtCFEM* genes at different time points post inoculation were normalized by actin gene and calibrated against that at 0 h. The assays were performed with three independent biological repetitions and three replicates each. A representative set of data are presented. Data are means ± standard error. Asterisks represent significant difference (*, *p* = 0.05, **, *p* = 0.01).

The transcriptional levels of secretory proteins are usually significantly induced during the infection process. To investigate whether the transcription of these eight *LtCFEM* genes is up-regulated during the infection, their relative transcription levels at infectious stages were examined by qRT-PCR. Our results showed that the expression profiles of all *LtCFEM* genes were classified mainly into three groups. The first group, such as *LtCFEM6*, was transcriptionally down-regulated during infection. The second group, i.e., *LtCFEM2*, maintained a relatively stable expression level, from approximately 0.5-fold to 1.5-fold. However, the expressions of third group including *LtCFEM1*, *LtCFEM3*, *LtCFEM4*, *LtCFEM5*, *LtCFEM7*, and *LtCFEM8* were significantly increased at the infectious stages. Additionally, the expressions of *LtCFEM1*, *LtCFEM4*, and *LtCFEM8* were induced at the early infection stage and then reached the highest level at 72 hpi. The transcript levels of *LtCFEM3* and *LtCFEM5* reached the apex at 48 hpi and then displayed a decreased tendency at 72 hpi. The expression of *LtCFEM7* was up-regulated drastically at 12 hpi and then maintained a relatively high expression level throughout the infection stages. These results showed that expressions of these eight *LtCFEM* genes were distinctly regulated during *L. theobromae* infection, indicating that these LtCFEM proteins may play different roles at different infection stages (Figure 4).

### Functional Validation of Predicted Signal Peptides of Eight LtCFEM Proteins

**FIGURE 5:**
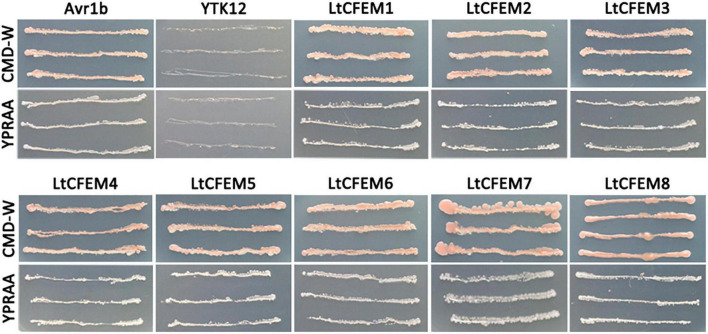
Functional validation of predicted signal peptides of eight LtCFEM proteins. The predicted signal peptides of LtCFEM1 to LtCFEM8 were able to guide the secretion of yeast invertase and therefore yeast transformants expressing *pSUC2:LtCFEM1* to *pSUC2:LtCFEM8* grew on YPRAA plate with raffinose as sole carbon source. The untransformed strain YTK12 grew neither on CMD-W medium nor on YPRAA medium. Transformants expressing *pSUC2:Avr1b* and *pSUC2:Mg87* were used as positive and negative controls, respectively. Yeast growth on CMD-W media showed an equal viability of these transformants.

An elegant yeast system, based on the requirement of invertase secretion for yeast cells to grow on sucrose or raffinose-containing media, was usually used to track the secretion of effector proteins (Oh et al., 2009; Gu et al., 2011; Tian et al., 2011; Fang et al., 2016). In this study, this genetic assay was exploited to corroborate the signal peptide function of total eight CFEM proteins. The predicted signal peptide sequence of each protein was fused in frame with yeast invertase whose signal peptide has been removed off. The resulting fusion construct was transformed into invertase secretion-deficient yeast strain YTK12. The signal peptide of each LtCFEM protein was able to guide the secretion of invertase, leading to yeast transformant grew on YPRAA media that contained raffinose as the sole carbon source. Also, the positive control, signal peptide of *Phytophthora sojae* Avr1b induce the secretion of invertase, and thereby, the correspondent yeast transformants grew on YPRAA media. By contrast, the negative control yeast strains expressing the signal peptide of *M. oryzae* Mg87 grew on CMD-W media but not on YPRAA plates (Figure 5). These results indicate the predicted signal peptides of these eight LtCFEM proteins are functional to direct these proteins to secretory pathways and these proteins are bona fide secreted proteins.

### The LtCFEM1 and LtCFEM4 Induced Local Yellowish Phenotype and Cell Death in *Nicotiana benthamiana* Leaves, Respectively

**FIGURE 6:**
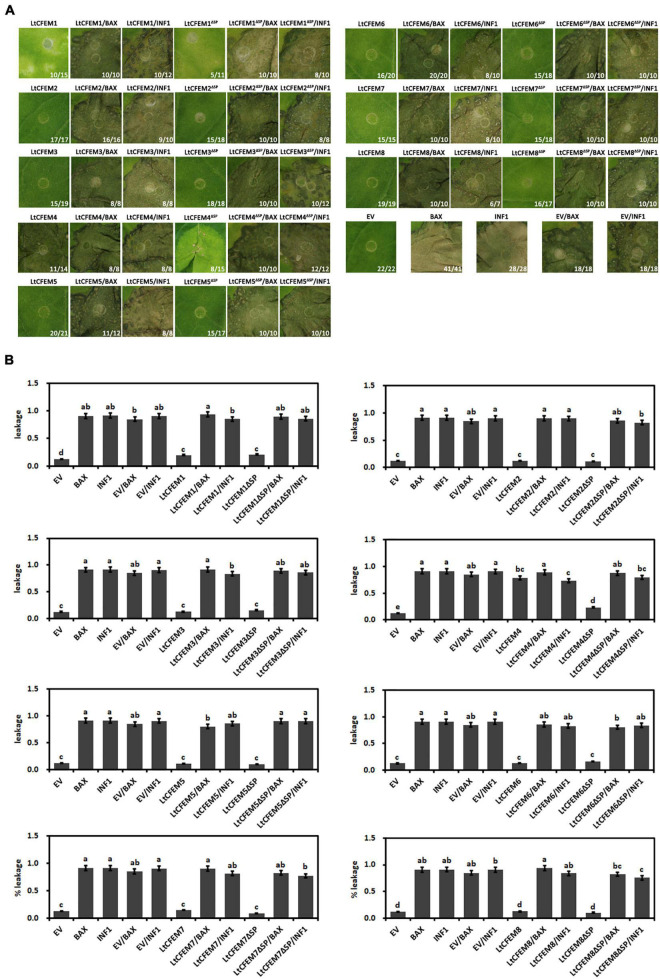
The LtCFEM1 and LtCFEM4 induced local yellowish phenotype and cell death in *Nicotiana benthamiana* leaves, respectively. **(A)** Transient expression of LtCFEM proteins in *N. bentamiana* leaves *via Agrobacterium*-mediated transformation. Photographs were obtained 9 days post agroinfiltration. Numbers, e.g., 11/14, indicate that 11 of 14 infiltrated leaves exhibited cell death or mottling phenotypes. *Agrobacterium tumefaciens* expressing EV (empty vector), BAX and INF1 were used as negative and positive controls, respectively. **(B)** Quantification of cell death *via* measuring the ion leakage of infiltrated *N. bentamiana* leaves. The ion leakage value between each LtCFEM protein with controls was compared and the significant differences among them were analyzed using the one-way analysis of variance (ANOVA) and least significant difference (LSD) tests (α = 0.05). Data are means ± standard error (SE) from three independent biological experiments. Letters a, b, c, and d indicate significant differences between different groups.

Previously identified CFEM domain-containing proteins were reported to affect cell death or chlorosis in *N. benthamiana* leaves (Zhu et al., 2017; Gong et al., 2020). To test whether the eight LtCFEM proteins possessed similar functions, these genes were expressed transiently in *N. benthamiana* leaves *via Agrobacterium*-mediated transformation. *Nicotiana benthamiana* leaves expressing BAX1 and INF1 proteins were used as positive controls, and the empty vector was used as the negative control. Among these proteins, transient expression of LtCFEM1 induced local yellowish lesions and LtCFEM4 induced cell death or chlorosis phenotypes in *N. benthamiana* leaves. However, other LtCFEM proteins did not exhibit any sign of programmed cell death in leaves (Figure 6A).

To determine whether the signal peptides of these LtCFEM proteins are required for the elicitor activity, the truncated form of LtCFEM protein (LtCFEM^ΔSP^) was expressed transiently in *N. benthamiana* leaves. Same to their full-length forms, LtCFEM proteins (except for LtCFEM1 and LtCFEM4) lacking signal peptide (LtCFEM^ΔSP^) also did not induce cell death in *N. benthamiana* leaves, which were further confirmed by the measured ion leakage values. Similar to the full-length form, LtCFEM1 truncated form LtCFEM1^ΔSP^ also triggered local yellowish phenotype in *N. benthamiana* leaves, and the LtCFEM4 truncated form LtCFEM4^ΔSP^, however, induced relatively attenuated chlorosis symptoms in comparison with full-length LtCFEM4 (Figure 6A). Taken together, it seems that the signal peptides of LtCFEM proteins (except for LtCFEM4) do not regulate their abilities or inabilities of inducing cell death in *N. benthamiana*.

Because ion leakage is positively correlated with leave cell death (Mittler et al., 1999; Fang et al., 2016), ion leakage of infiltrated leaves was measured to further quantify the cell death. In accordance with the observed cell death, leaves expressing LtCFEM1, LtCFEM1^ΔSP^, LtCFEM4, and LtCFEM4^ΔSP^ displayed higher levels of ion leakage in relation to negative control and other LtCFEM proteins (Figure 6B).

Because both the full-length and truncated forms of LtCFEM proteins (except for LtCFEM1 and LtCFEM4) could not trigger cell death or mottling phenotypes in *N. benthamiana* leaves, we investigated whether these proteins could suppress the cell death induced by BAX or INF1 elicitors. Hence, we transiently expressed the LtCFEM proteins 24 h before expressing the BAX or INF1 proteins using agroinfiltration. Nine days later, all *N. benthamiana* leaves expressing BAX or INF1 proteins displayed observable necrosis lesions, indicating that the LtCFEM proteins could not inhibit cell death arose by the two proteins, which was experimentally validated by the ion leakage measurement. Additionally, transient expression of the truncated form by agroinfiltration also did not inhibit the necrosis lesions caused by BAX or INF1 proteins, signifying that the signal peptide of LtCFEM protein did not influence its inability of suppressing the cell death caused by BAX or INF1 proteins in *N. benthamiana* (Figures 6A,B).

### The Localization of Eight LtCFEM Proteins in *Nicotiana benthamiana*

**FIGURE 7:**
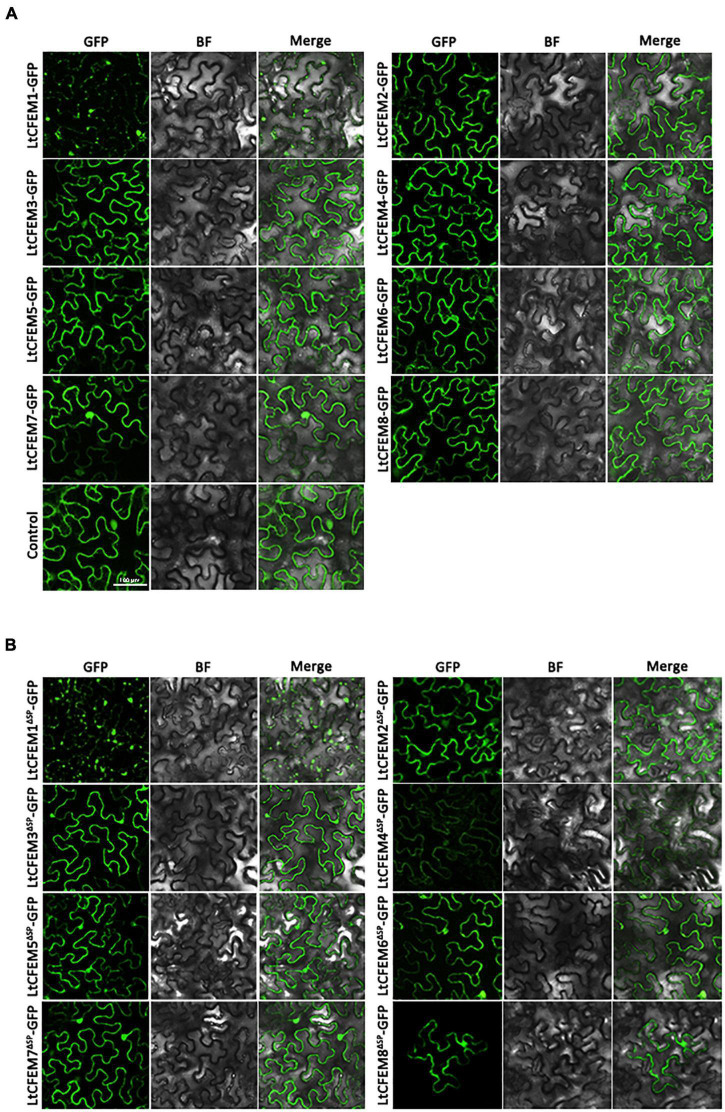
The subcellular localization of eight LtCFEM proteins in Nicotiana benthamiana. **(A)** The full-length ORF of each LtCFEM protein was co-expressed with GFP protein in *N. benthamiana*. The fluorescence of each LtCFEM-GFP protein was detected with confocal laser scanning microscope at the excitation wavelength of 488 nm. BF, Bright Field. **(B)** The truncated ORF of each LtCFEM protein was co-expressed with GFP protein in *N. benthamiana*. The fluorescence of each LtCFEMΔSP-GFP protein was detected with similar method described in **(A)**. ΔSP, proteins without signal peptide.

To gain insight into the molecular functions of the LtCFEM effector inside host cells, we engineered a fusion construct in which the functional C-terminal GFP was fused to mature LtCFEM protein. The fusion protein LtCFEM-GFP was transiently expressed by agroinfiltration in *N. benthamiana*. Fluorescence observation showed that the localizations of total fusion proteins were categorized mainly into two groups. The first group, including LtCFEM2-GFP, LtCFEM3-GFP, LtCFEM4-GFP, LtCFEM6-GFP, LtCFEM7-GFP, and LtCFEM8-GFP, were accumulated at cell nuclear and plasma membranes. The second group, i.e., LtCFEM5-GFP, was mainly concentrated at plasma membranes. Another group, LtCFEM1-GFP was gathered together and distributed in a puncta-like pattern (Figure 7A). To confirm the membrane localization of these LtCFEM proteins, we performed plasmolysis experiments on *N. benthamiana* leaves which transiently expressed these LtCFEM-GFP proteins. After treatment with the plasmolyticum agent, the green fluorescence was remained localized at the plasma membrane (Supplementary Figure 1A), suggesting these LtCFEM proteins, except for LtCFEM1, were nuclear and plasma membrane-localized proteins.

To further reveal whether the signal peptide of LtCFEM protein affects its subcellular localization, the truncated moiety of each LtCFEM protein was fused to C-terminal GFP fragment, followed by transient expression in *N. benthamiana*. Fluorescence detections showed that subcellular localizations of fusion proteins LtCFEMs^ΔSP^-GFP were localized into cellular nucleus and plasma membranes and the fluorescence intensity of LtCFEM4^ΔSP^-GFP was weaker than that of LtCFEM4-GFP (Figure 7B). Moreover, the membrane localization of these LtCFEM^ΔSP^-GFP proteins were further confirmed by the plasmolysis assays (Supplementary Figure 1B). Our results indicate that the signal peptide of these LtCFEM proteins do not have remarkable influence on their subcellular distributions.

### The Transcriptional Levels of *LtCFEM* Genes Under Different Temperatures

**FIGURE 8:**
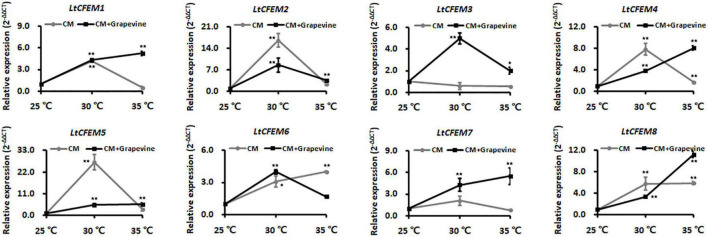
The transcription levels of *LtCFEM* genes under different temperature conditions. The mycelia of wild type CSS-01s were cultured in complete medium with or without 0.5% grapevine shoot powders and then harvested at 36 h post inoculation (hpi) for RNA extractions. The isolated RNA was reverse transcribed into cDNA for gene expression analyses. Relative transcription levels of *LtCFEM* genes were calculated using the 2^–ΔΔCT^ method. The relative transcript abundance of each *LtCFEM* gene was normalized by actin gene and calibrated against that at 25°. The assays were performed with three independent biological repetitions and three replicates each. A representative set of data are presented. Data are means ± standard error. Asterisks represent significant difference.

The grapevine canker disease caused by *L. theobromae* has been documented to be seriously affected by temperature, and secretory proteins were predicted to play significant roles in its virulence (Félix et al., 2019; Songy et al., 2019). To reveal whether the LtCFEM proteins were sensitive to environmental temperatures, transcriptional profiles of these genes were analyzed at different temperatures. The qRT-PCR analyses showed partial genes, including *LtCFEM1*, *LtCFEM2*, *LtCFEM4*, *LtCFEM5*, and *LtCFEM7*, displayed higher transcriptional levels at 30°C in comparison with that at 25°C and 35°C. Another two genes, *LtCFEM6 and LtCFEM8*, were transcriptionally up-regulated at both 30°C and 35°C when compared with that at 25°C. However, the expression of *LtCFEM3* was down-regulated at 30°C and 35°C temperatures (Figure 8).

For maintaining strictly uniform experimental conditions and to simulate the host environment, we deployed an artificial system by adding grapevine shoot powders into cultural media. When mycelia were cultured in complete media with 0.5% grapevine powder additives, the relative expression levels of *LtCFEM1*, *LtCFEM4*, *LtCFEM5*, *LtCFEM7*, and *LtCFEM8* were increased remarkably and reached highest level at 35°C (Figure 8), indicating that high temperature is conducive for their expression and infection.

Moreover, the transcript of *LtCFEM3* was also accumulated evidently at high temperature, especially at 30°C, when mycelia were cultured in media containing grapevine shoot powders. The significant differences indicated that *LtCFEM*3 was transcriptionally induced at high temperatures under artificial environment conditions, but its optimal expression temperature (30°C) was lower than other genes (*LtCFEM1*, *LtCFEM4*, *LtCFEM5*, *LtCFEM7*, and *LtCFEM8*) that were expressed preferentially at 35°C temperature (Figure 8).

Interestingly, under host-mimicking conditions, *LtCFEM6* was obviously down-transcribed at both 35°C and 25°C when compared with that at 30°C (Figure 8), suggesting that cool and hot waves do not favor its expression during the infection process.

### Alternative Splicing of mRNA Is Identified in *LtCFEM* Genes

In this study, when we amplified the transcripts of *LtCFEM* genes for transient expression through RT-PCR with total RNA extracted from wild-type mycelia, two *LtCFEM* genes *LtCFEM7* and *LtCFEM8* were found to be alternatively spliced, occasionally. As for *LtCFEM7* and *LtCFEM8* genes, two and three different transcript sizes were obtained, respectively, followed by confirmation *via* gel electrophoresis and sequencing (Supplementary Figure 2). However, for both differentially spliced genes, only the T1 transcript formed correct reading box and could be translated into complete full-length protein from the initiation codon to the termination codon, and translations of other transcripts were broken off, implying that two genes may play multiple roles in RNA and protein forms during mycelial development and fungal infection.

## Discussion

In previous studies, CFEM proteins including Pth11 (DeZwaan et al., 1999; Kou et al., 2017), WISH (Sabnam and Barman, 2017), BcCFEM1 (Zhu et al., 2017) and CCW14 (Moukadiri et al., 1997; Mrša et al., 1999; Mrša and Tanner, 1999) were found to be necessary for cell integrity, osmotic tolerance, invasion structure maturation and disease development in pathogenic or non-pathogenic fungi. However, their function in plant opportunistic pathogen has yet to be analyzed. In this study, we set out to identify and investigate the molecular functions of CFEM proteins in *L. theobromae* systemically. We focused on whether the CFEM proteins secreted by *L. theobromae* have an effect on plant immune responses and how the expression of these pathogenicity-related *LtCFEM* genes is affected by environmental factors such as temperature. Transcription detection of pathogenicity-related genes during fungal infection processes has become a widely used method for analyzing their potential pathogenic roles. In our research, six of eight *LtCFEM* genes were significantly up-expressed at the infectious stages, suggesting that these genes may function as important virulence factors. Further virulence tests of these genes were necessary for supporting the results of transcription profiles analyses.

The CFEM proteins were reported to be able to suppress cell death and induce chlorosis in tobacco leaves (Zhu et al., 2017; Gong et al., 2020; Wang et al., 2021). In this study, two proteins LtCFEM1 and LtCFEM4 were found to be involved in plant immune response because LtCFEM1 and LtCFEM4 triggered local yellowish phenotype and cell death lesion in *N. benthamiana* leaves, respectively. Moreover, it is documented that the signal peptides of effectors could affect their elicitor activities in phytopathogenic fungi such as *U. virens* (Fang et al., 2016), *M. oryzae* (Chen et al., 2013) and *C. graminicola* (Gong et al., 2020). In the current study, we found that LtCFEM4 triggered cell death lesion, and its truncated moiety LtCFEM4^ΔSP^ induced relatively attenuated chlorosis phenotype rather than cell death, suggesting signal peptide of LtCFEM4 is important for biological function. However, whether the signal peptides of other seven LtCFEM proteins affected their elicitor activities need further investigation. Moreover, the phenotype difference induced by LtCFEM4 and LtCFEM4^ΔSP^ may be related to their expression levels, because green fluorescence of LtCFEM4^ΔSP^-GFP was weakly detected, and LtCFEM4-GFP, however, was easily observed when both proteins were transiently expressed under similar conditions.

As mentioned above, the transcription levels of *LtCFEM1*, *LtCFEM3*, *LtCFEM4*, *LtCFEM5*, *LtCFEM7*, and *LtCFEM8* were elevated significantly at infection stages but only LtCFEM1 and LtCFEM4 caused plant immune response in *N. benthamiana* leaves. The differences can be attributed to that the pathogenicity-related proteins are important for fungal infection and disease development, but their significance during the infection were distinct and the extent of host responses vary upon their recognition by plant receptors.

As *L. theobromae* is regarded as an opportunistic plant pathogen (Chethana et al., 2016), and temperature with humidity have been accepted as two major environmental factors that seriously affect the severity of grapevine canker disease (Songy et al., 2019). In this study, we adopted an artificial system by adding exogenous grapevine shoot powders into cultural media to analyze the transcription levels of *LtCFEM* genes under different temperature. Similar experimental methods have also been used to identify and characterize pathogenicity related genes in proteomic and transcriptomic researches (Fernández-Acero et al., 2009; Cobos et al., 2010; Paolinelli-Alfonso et al., 2016). When the mycelia were cultured under host-mimicking state, infection-related genes including *LtCFEM1*, *LtCFEM4*, *LtCFEM5*, *LtCFEM7*, and *LtCFEM8* were preferentially expressed at higher temperature (35°C) rather than lower temperatures (25°C and 30°C), implying that high temperature may contribute to the disease development by *L. theobromae*. All the results substantiated by previous and current studies provide evidences for the claim that infection by *L. theobromae* was affected by the temperature factor.

Alternative splicing, a widely identified phenomenon identified in the genomes of plant, animal and fungi kingdoms, has been reported to be involved in ageing and longevity, health and disease, stress responses and plant immunity, plant-microbe interactions, and fungal virulence (Odenbach et al., 2007; Yang et al., 2014; Ruiz-Roldán et al., 2015; Kim et al., 2018; Rigo et al., 2019; Bhadra et al., 2020; Punzo et al., 2020). Two well-documented transcription factors, Con7 in *Fusarium oxysporum* with its homologs Con7p in *M. oryzae*, were identified to be necessary for the infection-related morphogenesis and pathogenesis (Odenbach et al., 2007; Ruiz-Roldán et al., 2015). Interestingly, among the two transcripts *Con7-1* and *Con7-2* in *F. oxysporum*, only the *Con7-1* was found to be involved in morphological maturation and fungal virulence. Although the biological functions of different transcripts have been examined in pathogenesis-related genes, alternative splicing was barely identified in secreted protein-coding genes. In this study, two secreted protein-coding genes *LtCFEM7* and *LtCFEM8* were found to be alternatively spliced by accident. Subsequent sequence analyses showed that for both genes, *LtCFEM7* and *LtCFEM8*, only the T1 transcript possess correct ORF from its initiation codon to termination codon, implying that these multiple transcripts may function in protein and RNA forms during the plant-pathogen interaction. However, because of the failure to generate gene-knockouts for these genes, we could not analyze the biological roles each transcript played during the whole life cycle of *L. theobromae*, and further functional investigations of different transcripts need to be explored in-depth.

In conclusion, we have systemically identified 14 CFEM proteins in *L. theobromae* and two of them, LtCFEM1 and LtCFEM4, were confirmed to be able to trigger local yellowish phenotype and cell death lesion in *N. benthamiana* leaves, respectively. Additionally, both two genes were transcriptionally up-regulated under high temperature. Interestingly, alternative splicing was identified in *LtCFEM7* and *LtCFEM8* genes, but the functions of distinct transcripts were still unrevealed. The results mentioned above will enhance our understanding of the regulatory mechanisms between grapevine-*L. theobromae* interaction. However, our finding also arises more important questions yet to be answered, for instance, why do effector genes need to be alternatively spliced? What are the molecular functions of each transcript? How did the high temperature affect the interactions between *Vitis vinifera*-*L. theobromae* through the CFEM proteins mediated signal pathways? Figuring out these issues will be of great significance to reveal the biological functions of secreted CFEM family proteins and get a better understanding of the opportunistically pathogenic mechanisms of this fungus.

## Data Availability Statement

The original contributions presented in the study are included in the article/Supplementary Material, further inquiries can be directed to the corresponding author.

## Author Contributions

JY and JP conceived and designed the experiments and wrote and revised the manuscript. JP, LW, and WZ performed the experiments. JP and XL analyzed the experiment data. QX, QZ, and XW assisted during molecular experiments and manuscript revision. All authors contributed to the article and approved the submitted version.

## Conflict of Interest

The authors declare that the research was conducted in the absence of any commercial or financial relationships that could be construed as a potential conflict of interest.

## Publisher’s Note

All claims expressed in this article are solely those of the authors and do not necessarily represent those of their affiliated organizations, or those of the publisher, the editors and the reviewers. Any product that may be evaluated in this article, or claim that may be made by its manufacturer, is not guaranteed or endorsed by the publisher.

## Footnotes

1
http://pfam.xfam.org/search#tabview=tab0

2
https://services.healthtech.dtu.dk/service.php?SignalP-5.0

3
https://services.healthtech.dtu.dk/service.php?TMHMM-2.0

4
http://weblogo.threeplusone.com/

5
http://www.sbg.bio.ic.ac.uk/phyre2/html/page.cgi?id=index

## Supplementary Material

Supplementary Figure 1 Plasmolysis investigation on *N. benthamiana* leaves. **(A)** Plasmolysis tests on *N. benthamiana* leaves which transiently expressed the full-length LtCFEM proteins. **(B)** Plasmolysis assays on *N. benthamiana* leaves which transiently express the truncated form of LtCFEM proteins. The *N. benthamiana* leaves were harvested 48 h post agroinfiltration and subsequently cut into small squares for treatment with 0.75 M sorbitol. After treatment for 15 min, slide carrying the samples were examined with a confocal laser scanning microscope at the excitation wavelength of 488 nm. BF, Bright Field. ^Δ^ SP, proteins without signal peptide.

Supplementary Figure 2 Alternative splicing of mRNA was identified in *LtCFEM7* and *LtCFEM8*. **(A)** Schematic representation of *LtCFEM7* alternative transcripts. Different transcripts were assured by sequencing. Black box and gray line marked the exon and intron regions, respectively. **(B)** Reverse-transcriptase polymerase chain reaction (RT-PCR) analyses of *LtCFEM7* transcripts. Letters T1 and T2 refer to the drawings depicted in **(A)**. **(C)** Multiple sequence alignments of *LtCFEM7* transcripts T1, T2 and genomic DNA. **(D)** Schematic representation of *LtCFEM8* alternative transcripts. **(E)** RT-PCR analyses of *LtCFEM8* transcripts. Letters T1, T2, and T3 refer to the drawings depicted in **(D)**. **(F)** Multiple sequence alignments of *LtCFEM8* transcripts T1, T2, T3 and genomic DNA.
