# Supplementary figures and images for: Systemic Identification and Functional Characterization of Common in Fungal Extracellular Membrane Proteins in Lasiodiplodia theobromae
Source: Front Plant Sci. 2021 Dec 20;12:804696. doi: 10.3389/fpls.2021.804696 (PMC8721227; doi:10.3389/fpls.2021.804696)

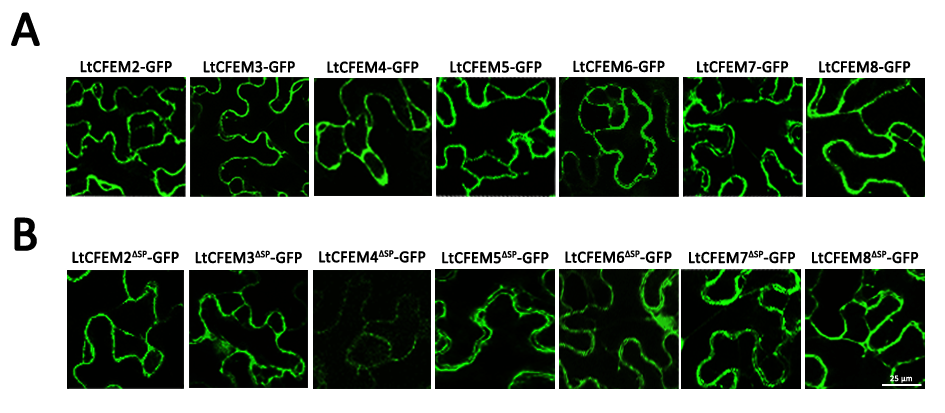

Supplement: Supplementary Figure 1 — Plasmolysis investigation on N. benthamiana leaves. (A) Plasmolysis tests on N. benthamiana leaves which transiently expressed the full-length LtCFEM proteins. (B) Plasmolysis assays on N. benthamiana leaves which transiently express the truncated form of LtCFEM proteins. The N. benthamiana leaves were harvested 48 h post agroinfiltration and subsequently cut into small squares for treatment with 0.75 M sorbitol. After treatment for 15 min, slide carrying the samples were examined with a confocal laser scanning microscope at the excitation wavelength of 488 nm. BF, Bright Field. Δ SP, proteins without signal peptide. [file Image_1.TIF]

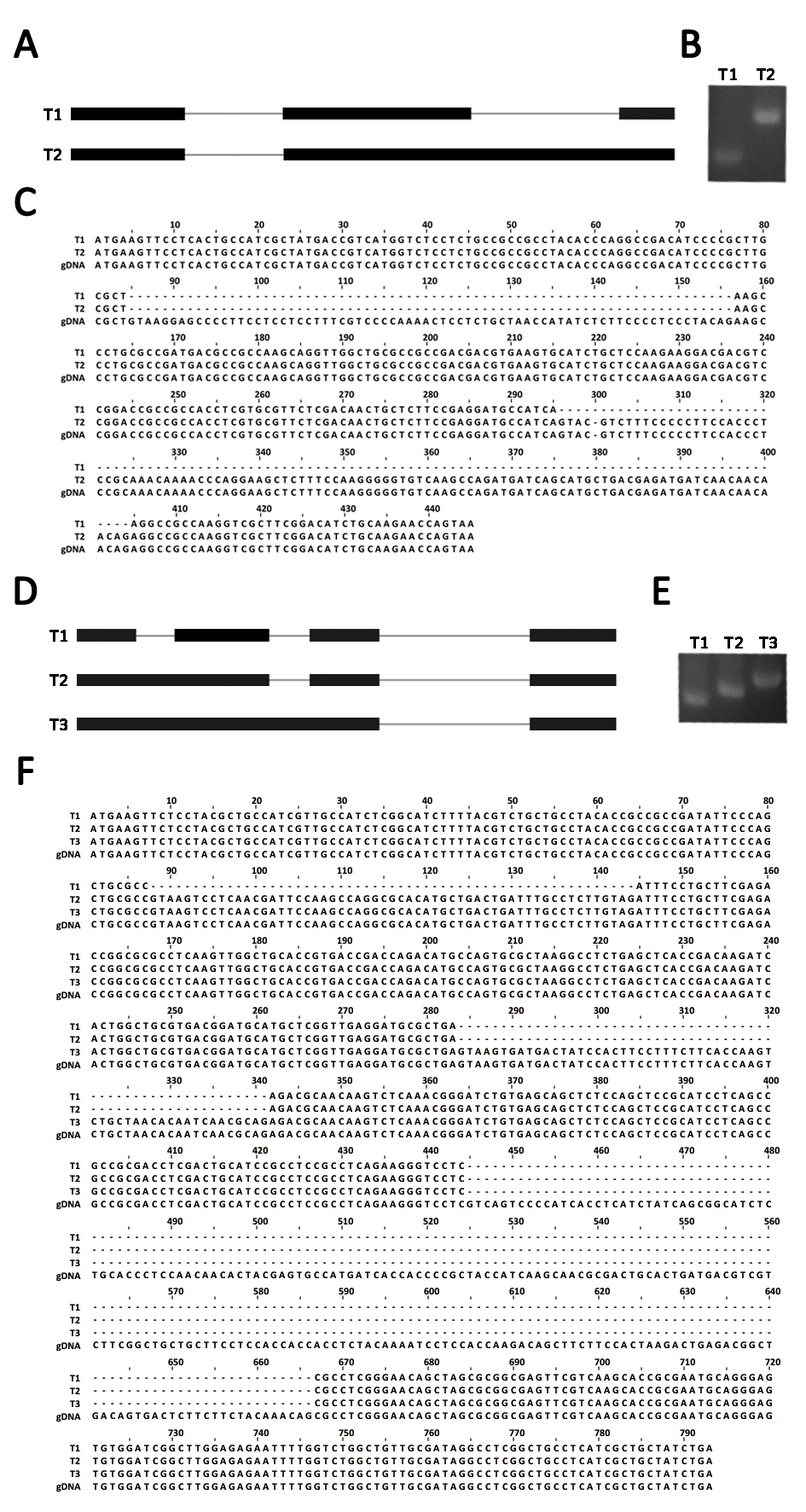

Supplement: Supplementary Figure 2 — Alternative splicing of mRNA was identified in LtCFEM7 and LtCFEM8. (A) Schematic representation of LtCFEM7 alternative transcripts. Different transcripts were assured by sequencing. Black box and gray line marked the exon and intron regions, respectively. (B) Reverse-transcriptase polymerase chain reaction (RT-PCR) analyses of LtCFEM7 transcripts. Letters T1 and T2 refer to the drawings depicted in (A). (C) Multiple sequence alignments of LtCFEM7 transcripts T1, T2 and genomic DNA. (D) Schematic representation of LtCFEM8 alternative transcripts. (E) RT-PCR analyses of LtCFEM8 transcripts. Letters T1, T2, and T3 refer to the drawings depicted in (D). (F) Multiple sequence alignments of LtCFEM8 transcripts T1, T2, T3 and genomic DNA. [file Image_2.TIF]
