# Supplementary material for: Systemic Identification and Functional Characterization of Common in Fungal Extracellular Membrane Proteins in Lasiodiplodia theobromae
Source: Front Plant Sci. 2021 Dec 20;12:804696. doi: 10.3389/fpls.2021.804696 (PMC8721227; doi:10.3389/fpls.2021.804696)
Supplement: Supplementary file 3 [file Table_1.DOCX]

**Supplementary Table 1. Primers used in this study.**

| Primer name | Sequence (5′-3′) | Use of primer |
| --- | --- | --- |
| Cfem1sp-f | CCGGAATTCATGCTGTCTTTTCTGCT | Used for the yeast complementary experiment of LtCFEM1 signal peptide |
| Cfem1sp-r | ATACTCGAGTTCCGGAAGGCTGCT |  |
| Cfem2sp-f | CGGAATTCATGAAGTCCTTCGCCG | Used for the yeast complementary experiment of LtCFEM2 signal peptide |
| Cfem2sp-r | ATACTCGAGGATGCAGGTTTGGCCA |  |
| Cfem3sp-f | CGGAATTCATGAAGTTCTCCACCG | Used for the yeast complementary experiment of LtCFEM3 signal peptide |
| Cfem3sp-r | ATACTCGAGAGGAATGGCGCAGG |  |
| Cfem4sp-f | CGGAATTCATGAAGACCTCCACCCTC | Used for the yeast complementary experiment of LtCFEM4 signal peptide |
| Cfem4sp-r | ATACTCGAGTTGGGCCGAGCTGAC |  |
| Cfem5sp-f | CGGAATTCATGAAGACCTCCTTCGC | Used for the yeast complementary experiment of LtCFEM5 signal peptide |
| Cfem5sp-r | ATACTCGAGGTTGAGCGAGCAGCT |  |
| Cfem6sp-f | CCGGAATTCATGCGCTTCTCTACAAT | Used for the yeast complementary experiment of LtCFEM6 signal peptide |
| Cfem6sp-r | ATACTCGAGGCATGATTGAGCGCA |  |
| Cfem7sp-f | CCGGAATTCATGAAGTTCCTCACTGC | Used for the yeast complementary experiment of LtCFEM7 signal peptide |
| Cfem7sp-r | ATACTCGAGGGGCTTAGCGCAAGC |  |
| Cfem8sp-f | CGGAATTCATGAAGTTCTCCTACGCT | Used for the yeast complementary experiment of LtCFEM8 signal peptide |
| Cfem8sp-r | TATCTCGAGAATGGCGCAGCTGGGA |  |
| Cfem1ex-f | CATGGTACCATGCTGTCTTTTCTGCTCCG | Used for the transient expression of LtCFEM1 and LtCFEM1^ΔSP^ |
| Cfem1ex-r | CATGGATCCCAGCTCTGCAAATATCATGG |  |
| Cfem1^ΔSP^ex-f | CATGGTACCATGTTTGATTTCAGCAGCC |  |
| Cfem2ex-f | CATGGTACCATGAAGTCCTTCGCCGTCCT | Used for the transient expression of LtCFEM2 and LtCFEM2^ΔSP^ |
| Cfem2ex-r | CATGGATCCCAGAGCGAGCAGCATGGC |  |
| Cfem2^ΔSP^ex-f | CATGGTACCATGCCTGAGTGTGGCCAAA |  |
| Cfem3ex-f | CATGGTACCATGAAGTTCTCCACCGCTGT | Used for the transient expression of LtCFEM3 and LtCFEM3^ΔSP^ |
| Cfem3ex-r | CATGGATCCGAGGAGGAGCATGACGG |  |
| Cfem3^ΔSP^ex-f | CATGGTACCATGACCGCCGACATCCC |  |
| Cfem4ex-f | CATGGTACCATGAAGACCTCCACCCTCCT | Used for the transient expression of LtCFEM4 and LtCFEM4^ΔSP^ |
| Cfem4ex-r | CATGGATCCGTTCCCCAAAGTGAAAATCT |  |
| Cfem4^ΔSP^ex-f | CATGGTACCATGAGCTCGGCCCAATCG |  |
| Cfem5ex-f | CATGGTACCATGAAGACCTCCTTCGCT | Used for the transient expression of LtCFEM5 and LtCFEM5^ΔSP^ |
| Cfem5ex-r | CATGGATCCCAAGCCGGCGACAGC |  |
| Cfem5^ΔSP^ex-f | CATGGTACCATGATCTCCGACCTCCCCA |  |
| Cfem6ex-f | CATGGTACCATGCGCTTCTCTACAATCTC | Used for the transient expression of LtCFEM6 and LtCFEM6^ΔSP^ |
| Cfem6ex-r | CATGGATCCCAGAGCACCCATAAGACCA |  |
| Cfem6^ΔSP^ex-f | CATGGTACCATGCAGCTGCTCCCCCA |  |
| Cfem7ex-f | CATGGTACCATGAAGACCTCCACCCTCCT | Used for the transient expression of LtCFEM7 and LtCFEM7^ΔSP^ |
| Cfem7ex-r | GGATCCGTTCCCCAAAGTGAAAATCT |  |
| Cfem7^ΔSP^ex-f | CATGGTACCATGCCCGCTTGCGCTAA |  |
| Cfem8ex-f | CATGGTACCATGAAGTTCTCCTACGCT | Used for the transient expression of LtCFEM8 and LtCFEM8^ΔSP^ |
| Cfem8ex-r | GGATCCGATAGCAGCGATGAGGC |  |
| Cfem8^ΔSP^ex-f | CATGGTACCATGACCGCCGCCGATATT |  |
| Cfem1qrt-f | GCAGCGGCGATGATATGATG | Used for the transcription profile analyses of *LtCFEM1* |
| Cfem1qrt-r | GCAGTGGGAGTAACGGACAT |  |
| Cfem2qrt-f | CCGTGGCTCAGACTCTTTC | Used for the transcription profile analyses of *LtCFEM2* |
| Cfem2qrt-r | GTCGCTAGTGGCAATGGAA |  |
| Cfem3qrt-f | CCTTGCTGCCGAGGTCATC | Used for the transcription profile analyses of *LtCFEM3* |
| Cfem3qrt-r | GTGGTCTTGCCGCTGTTCA |  |
| Cfem4qrt-f | ACTCAGCACCGTCGTCTCT | Used for the transcription profile analyses of *LtCFEM4* |
| Cfem4qrt-r | CGTCTCGCCACCAACAGTAG |  |
| Cfem5qrt-f | GCCTACTCCCAGGACATCT | Used for the transcription profile analyses of *LtCFEM5* |
| Cfem5qrt-r | GAGAGACGAGCAACCATCAC |  |
| Cfem6qrt-f | CGCTTCTCTACAATCTCTACCA | Used for the transcription profile analyses of *LtCFEM6* |
| Cfem6qrt-r | CAAATGCACTCGACATCAAGA |  |
| Cfem7qrt-f | ACGACGTGAAGTGCATCTG | Used for the transcription profile analyses of *LtCFEM7* |
| Cfem7qrt-r | CATCCTCGGAAGAGCAGTTG |  |
| Cfem8qrt-f | ACCGTGACCGACCAGACAT | Used for the transcription profile analyses of *LtCFEM8* |
| Cfem8qrt-r | CGCATCCTCAACCGAGCAT |  |
| Actinqrt-f | CCAAGTCCAACCGTGAGAAG | Used for the transcription profile analyses of internal control |
| Actinqrt-r | GAAGCGTACAGCGACAGAAC |  |
